# Multivariate Assessment and Risk Ranking of Pesticide Residues in Citrus Fruits

**DOI:** 10.3390/foods12132454

**Published:** 2023-06-22

**Authors:** Jelena Radulović, Milica Lučić, Aleksandra Nešić, Antonije Onjia

**Affiliations:** 1 Anahem Laboratory, Mocartova 10, 11160 Belgrade, Serbia; 2 Innovation Center of the Faculty of Technology and Metallurgy, Karnegijeva 4, 11120 Belgrade, Serbia; 3 Vinča Institute of Nuclear Sciences, University of Belgrade, Mike Alasa 12-14, 11351 Belgrade, Serbia; 4 Faculty of Technology and Metallurgy, University of Belgrade, Karnegijeva 4, 11120 Belgrade, Serbia

**Keywords:** LC-MS/MS, GC-MS/MS, QuEChERS, health risks, Monte Carlo simulation, sensitivity analysis

## Abstract

Pesticides are extensively used in the cultivation and postharvest protection of citrus fruits, therefore continuous monitoring and health risk assessments of their residues are required. This study aimed to investigate the occurrence of pesticide residues on citrus fruits and to evaluate the acute and chronic risk for adults and children. The risk ranking of twenty-three detected pesticides was carried out according to a matrix ranking scheme. Multiple residues were detected in 83% of 76 analyzed samples. In addition, 28% contained pesticides at or above maximum residue levels (MRLs). The most frequently detected pesticides were imazalil, azoxystrobin, and dimethomorph. According to the risk ranking method, imazalil was classified in the high-risk group, followed by prochloraz, chlorpyrifos, azinphos-methyl, tebufenpyrad, and fenpiroximate, which were considered to pose a medium risk. The majority of detected pesticides (74%) posed a low risk. The health risk assessment indicated that imazalil and thiabendazole contribute to acute (HQa) and chronic (HQc) dietary risk, respectively. The HQc was negligible for the general population, while the HQa of imazalil and thiabendazole exceeded the acceptable level in the worst-case scenario. Cumulative chronic/acute risk (HIc/HIa) assessment showed that chronic risk was acceptable in all samples for children and adults, while the acute risk was unacceptable in 5.3% of citrus fruits for adults and 26% of citrus fruits for children. Sensitivity analyses indicated that the ingestion rate and individual body weight were the most influential risk factors.

## 1. Introduction

Citrus fruits are among the most cultivated and consumed fruits in the world due to their pleasant taste, flavor, and aroma. In addition, they are sources of vitamins (C, A, E, and B), minerals, antioxidants, and dietary fibers. Citrus belong to the genus *Citrus* L. in the family *Rutaceae* and include oranges, lemons, mandarins, grapefruits, limes, citron, kumquats, tangelos, hybrids, and others [1,2,3]. The top-producing countries in 2021 were China, Brazil, India, and Mexico [4]. Although Asia produces the most citrus (51%), the most considerable quantities are exported from the Mediterranean (52% of citrus exports originate from this region). The top exporting countries in 2021 were Spain, South Africa, Turkey, and Egypt [5].

Citrus fruits are vulnerable to numerous diseases and parasites during fruit development and later storage, so pesticides are employed in various stages of fruit development and after harvest. Modern agricultural production is impossible without pesticides, although these chemicals pose a risk to humans and other non-target organisms [6,7]. Pesticides can be classified based on the type of pests they kill as insecticides, herbicides, fungicides, and rodent repellants. After the application, large amounts of pesticides can pass into water and soil, where they remain for different periods depending on the amount applied, type of soil, type of pesticide, application of fertilizers, and other factors [8]. Food is the main route of pesticide intake, where fruits and vegetables represent the riskiest foods due to the large amount of pesticide applied and because they are usually consumed fresh. Pesticide residues taken through food consumption are five times higher than those introduced through water and air [9,10]. For the pre- and postharvest protection of citrus fruits, numerous pesticides are applied. Due to the extensive use of pesticides, citruses are among the most pesticide-contaminated food [11]. The most often identified in citrus fruits are insecticides and acaricides [12], while the highest residue concentrations are from fungicides applied close to harvest time or at postharvest [10]. Some of the most detected are chlorothalonil (fungicide), daminozide (herbicide) [7,8,10], chlorpyrifos (insecticide) [8,10,12] and etoxazole (insecticide) [12] and post-harvest fungicides imazalil and thiabendazole [13].

The most commonly used methods for the multiresidue pesticide analysis of fruits and vegetables are gas chromatography (GC) and liquid chromatography (LC) [14,15]. Better sensitivity, separation, and identification abilities are achieved when GC and LC are coupled with the mass detection technique, particularly tandem mass spectrometry (MS/MS) [15]. The high selectivity provided by LC-MS/MS and GC-MS/MS allows for the determination of many pesticides at trace levels in various matrices in one analytical run [16,17]. The LC-MS/MS is typically used to detect polar and semipolar compounds, while the GC-MS/MS is generally used to detect nonpolar and semipolar pesticides [18].

The highest concentration of pesticides allowed in crops is defined as the maximum residue limit (MRL). The legislation of many countries and legal directives of different organizations established MRL values for various food commodities. The most important are MRLs adopted by the Codex Alimentarius Commission (CAC) [19], the European Union (EU) [20], the United States of America (USA) [21,22], China [23], Russia, Canada, Australia, Japan, New Zealand, Hong Kong, India, Malaysia, Indonesia, and South Korea [24]. The MRL value represents the maximum allowed concentration of a pesticide in food, but even products that exceed the MRL value are sometimes consumed [25].

Pesticides and their metabolites can have health implications, such as respiratory problems, cancer, genotoxicity, neurotoxicity, endocrine disrupting toxicity, etc. Hence, properly controlling pesticide application in fields and evaluating pesticide residue risk assessments on human health is essential [26,27,28]. Current findings indicate no safe exposure level for certain pesticides in food, for example, chlorpyrifos [29]. This pesticide is banned in the EU [29] and USA [30] but not in other countries, so it can still be found in citrus fruits [8]. Thus, monitoring pesticide residues in food is necessary due to many possible health issues [31,32]. Dietary health risk assessments of pesticide residues in food are based on combining consumption and contamination data. For this evaluation, the estimated daily intake (EDI) is compared with acceptable daily intake (ADI)—chronic risk and acute reference dose (ARfD)—acute risk [10].

Important multivariate statistic methods in food quality and safety analysis include principal component analysis (PCA) and hierarchical cluster analysis (CA). The latter method uses a dendrogram to present distances between samples [33,34].

Additionally, the residual risk of each detected pesticide is ranked using a matrix ranking score [35]. Finally, the total score of the pesticide is calculated based on the toxicity score and exposure score. In this way, the intake risks of pesticides are categorized and prioritized [25]. Uncertainty is generally associated with health risk assessment due to insufficient data about some parameters or their variability. Hence, deterministic health risk assessment can be coupled with Monte Carlo simulation to obtain more accurate health risk estimation [36,37]. The Monte Carlo simulation is a probabilistic approach recommended by the USEPA for risk assessments [38].

This study aimed to assess the concentrations of pesticide residues in different citrus fruits imported to Serbia. As a result, deterministic and probabilistic health risks for consumers have been estimated. Additionally, the exposure risks of each of the detected pesticides were ranked using a matrix ranking method.

## 2. Materials and Methods

### 2.1. Reagents and Chemicals

The pesticide standards were purchased from Restek (Bellefonte, Pennsylvania, USA) and Lab Instruments (Castellana Grotte, Italy). HPLC-MS-grade acetonitrile, methanol, formic acid, and acetic acid were obtained from Lachner (Neratovice, Czech Republic). Sodium chloride, magnesium sulfate anhydrous, disodium hydrogen citrate sesquihydrate (C_6_H_8_Na_2_O_8_), and trisodium citrate dihydrate (C_6_H_5_O_7_Na_3_ × 2H_2_O) were obtained from Lachner (Czech Republic). The graphitized carbon black (GCB), C18 sorbent, and primary and secondary amines (PSA) were supplied from Sigma-Aldrich (St Louis, Missouri, USA).

The selected citrus fruits were purchased from local markets in Belgrade, Serbia, in February 2022, and a total of 76 samples were analyzed (28 oranges, 26 lemons, 17 tangerines, and 5 grapefruits). Each fruit sample was analyzed as the whole fruit following European Union Guidelines Regulation, (EC) No. 396/2005 Annex [20]. During the survey, fruits were stored in fridges in labeled plastic bags at 4 °C until analysis.

### 2.2. Sample Preparation

Citrus samples were grounded in a blender as a whole fruit. The extractions of pesticide residues were performed according to the QuEChERS protocol (Quick, Easy, Cheap, Effective, Rugged, and Safe) [39]. According to the protocol, 10 g of the sample was weighed in a plastic tube, and 10 mL of acetonitrile was added. Then, four salts were added (4 g of MgSO_4_, 1 g of NaCl, 1 g of C_6_H_5_O_7_Na_3_ × 2H_2_O, and 0.5 g of C_6_H_8_Na_2_O_8_) to each tube. All samples were well vortexed and centrifuged at 3200 rpm for 5 min. Next, the upper acetonitrile layer was transferred to a tube that contained 900 mg of MgSO_4_, 150 mg of primary and secondary amines, and 150 mg of C18, which enabled the removal of waxes from the sample. The tube was then centrifuged again at 3200 rpm for 5 min, allowing the layers to be separated. Finally, the clear upper acetonitrile layer is taken to be analyzed for pesticide residues.

### 2.3. Instrumental Analysis

GC-MS/MS analysis of pesticides was carried out using a Thermo Trace 1310 GC coupled to a TSQ 8000 Evo mass spectrometer operating with an EI source. The system was equipped with a TR Pesticide II column (30 m × 0.25 mm × 0.25 µm, Thermo Scientific, Waltham, Massachusetts, USA), a split/splitless injector operating in splitless mode, and a Thermo Scientific TriPlus AS autosampler. The volume of injection in the splitless mode was 1.0 µL. The PTV inlet temperature increased from 75 °C (initial hold: 1 min) up to 330 °C (in 2 min). The oven temperature program was 60 °C for 2.3 min, increasing to 90 °C at a rate of 25 °C/min and held for 1.5 min, then increasing to 180 °C at a rate of 25 °C/min and held for 0 min, then increasing to 280 °C at a rate of 5 °C/min and held for 0 min, and finally increased to 300 °C at a rate of 10 °C/min and held for 5 min. The carrier gas was helium with a flowrate of 1.20 mL/min. The MS transfer line temperature was 250 °C, and the ion source temperature was 300 °C.

LC-MS/MS analysis of pesticides was carried out using a Thermo Scientific (San Jose, California, USA) Accela HPLC system coupled to a triple-quadrupole mass spectrometer (Thermo Scientific TSQ Quantum Access MAX model), operating with a heated electrospray ionization (HESI) source. The separation was performed on a Accucore aQ C18 HPLC Column (2.1 mm × 100 mm, 2.6 μm, Thermo Scientific). The mobile phase consists of (A) water containing 0.1% formic acid and 5 mM of HCOONH_4_ and (B) methanol containing 0.1% formic acid and 5 mM of HCOONH_4_. Gradient elution was performed as follows: start with 100% eluent A; then A:B = 30:70 at 7 min; then 100% eluent B at 9 min, holding for 3 min; then back to 100% eluent A at 12.5 min; and keep for 4 min. The column temperature was maintained at 40 °C. The flowrate was 0.3 mL/min with an injection volume of 10 µL.

### 2.4. Quality Assurance/Quality Control

To validate the method of analysis, the following parameters were evaluated: linearity, matrix effect, selectivity, sensitivity, accuracy, and precision. The European Union SANTE/11312/2021 regulatory guidelines were followed [40]. Two calibration curves were constructed by analyzing standards in the solvent and standards in a matrix. For the HPLC-MS/MS analysis, standard solutions were prepared in acetonitrile and matrix extract at concentration levels of 5, 10, 25, 50, 100, 150, and 200 µg/L, and for the GC-MS/MS analysis, standard solutions were similarly prepared in acetonitrile and matrix extract at concentration levels of 5, 10, 25, 50, 100, 150, and 200 µg/L. The linearity was accepted if the correlation coefficients (r^2^) from the calibration curves were equal to or higher than 0.99. The matrix effect (*ME*) was calculated according to the following equation:

(1) $$ME\;(in\;\%)=\left(\frac{R_{std}\;in\;matrix\;extract}{R_{std}\;in\;solvent}\right)\times 100$$

where *R_std_* is the detector response. The selectivity of the method was examined by analyzing the solvent blank and control citrus sample to the pesticide mix standard. The obtained chromatograms were compared to separate the analytes from the noise and other matrix substances. The sensitivity was evaluated by the limit of detection and limit of quantification (LOQ) for spiked samples. The limit of detection is defined as the lowest pesticide concentration in the fruit samples that gave a response 3 times greater than the signal-to-noise (S/N) ratio, while the LOQ is the lowest spike level of the validation meeting the method performance acceptability criteria.

### 2.5. Risk-Ranking Score

The residual risk score for detected pesticides is calculated based on the matrix ranking scheme developed by the Veterinary Residues Committee of the UK [35]. The residual risk score (*S*) of pesticides is calculated according to Equation (2):

(2) $$S=\left(A+B\right)\times \left(C+D+E\right)\times F$$

where *A* is the toxicity score determined based on *LD*_50_ acquired from the World Health Organization [41], *B* is the toxic potency score determined based on *ADI* obtained from the EU Pesticide database [42], JMPR database [43] or Pesticide Properties DataBase (PPDB) [44], *C* is the score of the citrus fruits proportion in total diet acquired from Household budget survey data in Serbia for 2021 [45], *D* is the score of the frequency of dosing with a particular pesticide, *E* is the score of the evidence of high exposure groups (this score is set at 3 due to no sufficient data on high exposure groups), and *F* is the score of the detectable pesticide residue level determined based on maximum residue level (*MRL*) acquired from the EU Pesticide database [20], JMPR database [43] or PPDB [44]. The frequency of dosing (*FOD*) is calculated according to Equation (3):

(3) $$FOD=\frac{N}{T}\times 100$$

where *N* is the number of times in days the pesticide was used during plantation, and *T* is the number of growth days. The score for the residual level (*F*) is determined based on the MRL values [20] using Equation (4):

(4) $$F=\frac{{F_{0}\times 1+F}_{1}\times 2+F_{2}\times 3+F_{3}\times 4}{n}$$

where *F*_0_ is the number of free-pesticide samples, *F*_1_ is the number of samples with pesticide residue below 1 *MRL*, *F*_2_ is the number of samples with pesticide residue ≥1–10 *MRL*, and *F*_3_ is the number of samples with pesticide residue ≥10 *MRL* [46]. The definition and individual scores for A–F are given in Table S1. Summary tables with *LD*_50_, *ADI*, *MRL*, and assigned scores are given in Tables S2 and S3.

### 2.6. Dietary Exposure Assessment

The chronic exposure risk *(*%*ADI*), i.e., the chronic hazard quotient (*HQc*) is calculated as the national estimated daily intake (*NEDI*, mg/kg *bw*) divided by the acceptable daily intake (*ADI*, mg/kg *bw*):

(5) $$NEDI=\frac{STMR\times Fi}{bw}$$

(6) $$HQc=\frac{NEDI}{ADI}$$

(7) %ADI=HQc×100

where *STMR* represents the median value of pesticide residue in one sample, *Fi* (kg/day) is the average daily citrus consumption per capita in Serbia, and *bw* is the average body weight (kg).

The acute (short-term) intake risk (%*ARfD*), i.e., acute hazard quotient (*HQa*) is calculated as the international estimation of short-term intake (*IESTI*, mg/kg *bw*) divided by the acute reference dose of pesticide (*ARfD*, mg/kg *bw*). For different types of citrus fruits tree, versions of equations were used for the calculation of *IESTI* (Equations (8a–c)) [47]:

(8a) $$Case\;1:Ue<25\;g\;\;\;\;\;\;\;\;IESTI=\frac{LP\times HR}{bw}$$

(8b) $$Case\;2a:\;25\;g\leq Ue<LP\;\;\;\;IESTI=\frac{Ue\times HR\times v+(LP-Ue)\times HR}{bw}$$

(8c) $$Case\;2b:\;Ue>LP\;\;\;\;\;\;\;\;IESTI=\frac{Ue\times HR\times v}{bw}$$

The *HQa*, i.e., %*ARfD* is calculated using the following equation:

(9) $$HQa=\frac{IESTI}{ARfD}$$

(10) %ARfD=HQa×100

where *LP* is the highest large portion provided (97.5th percentile of eaters), in kg of food per day; *HR* is the highest residue in a composite sample of the edible portion of the unit; *Ue* is the weight of the edible portion of the unit, in kg; *bw* is the average body weight for a population age group, in kg; and *v* is the between-individual variability factor [48].

The cumulative chronic (*HIc*) and cumulative acute risk (*HIa*) are calculated according to Equation (11):

(11) HI=∑HQ

The cumulative chronic or acute risk represents the sum of *HQc* or *HQa* of each detected pesticide in a single citrus fruit. When *HQc*, *HQa*, or *HI* are higher than 1 (i.e., %*ADI*, %*ARfD*, or %*HI* are higher than 100%), there is a greater probability of adverse health effects due to the presence of pesticides. In this case, the food items may not be acceptable for consumption.

### 2.7. Monte Carlo Analysis

The chronic risk uncertainty and sensitivity were estimated by Monte Carlo simulation using Oracle Cristal Ball software. The repetition number of each calculation was set at 100,000. The lognormal distribution of the residue concentrations and ingestion rate, normal distribution of body weights, and uniform distribution of other variables were assumed.

## 3. Results and Discussion

### 3.1. Method Validation

Good linearity for all tested pesticides was demonstrated by the coefficients of correlation (r^2^) in the range of 0.9932 to 0.9972. The detection limits ranged from 1 µg/kg (imidacloprid) to 3 µg/kg (imazalil), and all LOQs were ≤10 µg/kg. For the ME evaluation, two fruit matrices (lemons and oranges) were enriched with pesticide standards at 5–200 µg/kg concentrations. The matrix effect did not significantly affect the suppression or enhancement of the signal (ME < 20%). Spiked samples of the fruit extracts with pesticide concentrations of 10 and 100 µg/kg were analyzed six times to obtain recoveries that ranged from 76 to 118%. The recovery of pesticides at the lower concentration level ranged from 76% (metolachlor) to 118% (acetamiprid), while at the higher concentration level, it ranged from 89% (azoxystrobin) to 112% (chlorpyrifos). The relative standard deviations (RSDs) vary from 4.3% (propiconazole) to 19% (boscalid) for pesticides spiked at 10 µg/kg concentration levels and from 2.9% (metolachlor) to 14% (dimethomorph) for pesticides spiked at 100 µg/kg concentration levels.

### 3.2. Occurrence of Pesticide Residues in Citrus Samples

The analysis of 76 citrus fruit samples revealed the presence of 23 different pesticides (Table S4). The vast majority of citrus fruits had multiresidues (two pesticides in 18 fruits, three pesticides in 20 fruits, four pesticides in 16 fruits, five pesticides in 6 fruits, six pesticides in 1 fruit, and seven pesticides in 2 fruits). Thirteen samples had a single pesticide residue. In total, 9 of the 23 detected pesticide residues were not approved by European legislation (propiconazole, carbendazim, metolachlor, azinphos-methyl, imidacloprid, prochloraz, chlorpyrifos, picoxystrobin, and fluazifop) [49]. Unapproved pesticides were detected in 29% of the analyzed citrus samples. The most detected pesticides were fungicides: imazalil (88% of samples), azoxystrobin (41% of samples), dimethomorph (37% of samples), boscalid (33% of samples), pyrimethanil (26% of samples), and thiabendazole (24% of samples).

The study of Fernandez et al. (2000) [50] also showed that the most frequently detected pesticides in oranges from Spain were imazalil (74%) and thiabendazole (14%). Similar results were found in several previous studies. In the study of Jurak et al. (2021) [51], imazalil was the most frequently detected pesticide in citrus fruits, followed by chlorpyrifos (studies from Croatia and Slovenia). In different fruits from South America, the three most frequently detected pesticides were thiabendazole (29%), imazalil (25%), and chlorpyrifos (17%) [52]. Similarly, in lemons from Turkey, the most detected pesticide was chlorpyrifos-methyl (17%) [53]. On the other hand, the research of Li et al. (2020) [10] found that in citrus grown in China, the most common pesticides were the fungicides prochloraz (26%) and carbendazim (21%), the insecticides profenofos (18%) and acetamiprid (18%), the acaricides spirodiclofen (17%) and propargite (16%), and the fungicide tebuconazole (16%).

**Figure 1:**
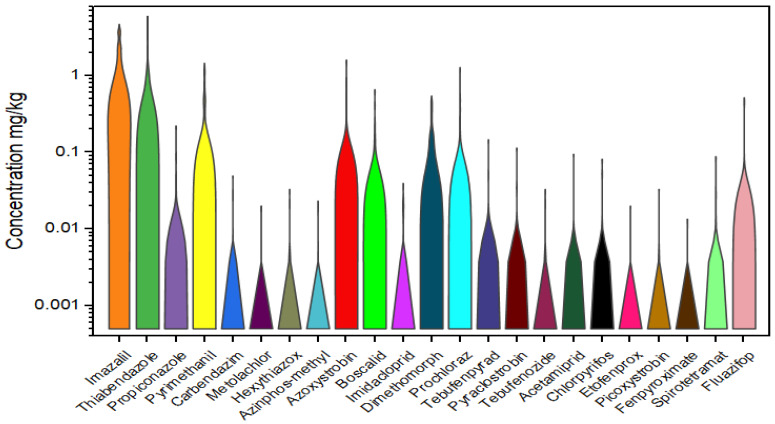
Violin plots illustrating the distribution of pesticides in citrus fruits.

Violin plots were used to show the full distribution of pesticide residues in the analyzed samples (Figure 1). The pesticide concentrations are displayed using a log scale. The height of the violin plots describes the range of the residue concentration and suggests that the largest ranges were for imazalil, thiabendazole, pyrimethanil, azoxystrobin, prochloraz, boscalid, dimethomorph, and fluazifop. Acetamiprid, etofenprox, picoxystrobin, and fenpyroximate were the least frequently detected pesticides (each pesticide residue detected only in one sample).

The highest pesticide concentration detected was for the pesticide thiabendazole in one orange sample (5.9 mg/kg). However, this concentration is not above the allowed MRL value for thiabendazole. Some of the pesticides were above the MRL values, such as imazalil in one grapefruit, propiconazole in three samples of lemons, azinphos-methyl in two lemons, dimethomorph in eight mandarins, prochloraz in two mandarins and two oranges, chlorpyrifos in one tangerine and one lemon, picoxystrobin in one tangerine, and fluazifop in two oranges. So, the MRL values of pesticide residues were exceeded in 21 samples (28%).

### 3.3. Multivariate Analysis

The correlation matrix of analyzed pesticide residues is presented in Table 1 (0.05 levels of significance). A strong positive correlation is observed between picoxystrobin and etofenprox (r = 0.999), chlorpyrifos and acetamiprid (r = 0.830), pyraclostrobin and imidacloprid (r = 0.743), tebufenozide and pyraclostrobin (r = 0.541), and dimethomorph and thiabendazole (r = 0.529), suggesting a common origin. A moderate positive correlation existed between acetamiprid and boscalid (0.490), chlorpyrifos and boscalid (0.388), tebufenozide and imidacloprid (0.367), etofenprox and tebufenpyrad (0.331), picoxystrobin and tebufenpyrad (0.331), azoxystrobin and azinphos-methyl (0.319), acetamiprid and dimethomorph (0.312), and tebufenpyrad and imidacloprid (0.302). Correlations between other pesticide residues were insignificant, and in most cases, it was negative.

**Figure 2:**
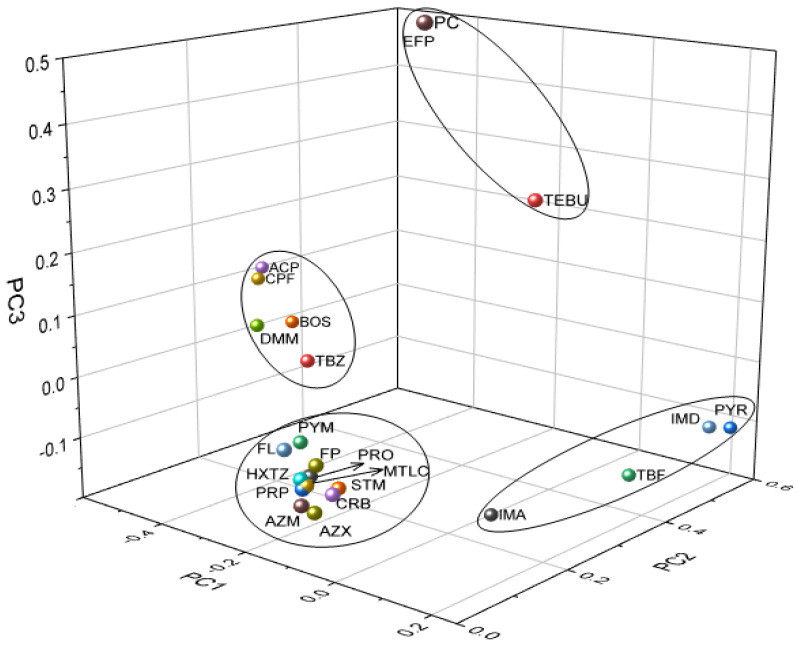
Principal component analysis (PCA) analysis of imazalil (IMA), thiabendazole (TBZ), propiconazole (PRP), pyrimethanil (PYM), carbendazim (CRB), metolachlor (MTLC), hexythiazox (HXTZ), azinphos-methy (AZM), azoxystrobin (AZX), boscalid (BOS), imidacloprid (IMD), dimethomorph (DMM), prochloraz (PRO), tebufenpyrad (TEBU), pyraclostrobin (PYR), tebufenozide (TBF), acetamiprid (ACP), chlorpyrifos (CPF), etofenprox (EFP), picoxystrobin (PC), fenpyroximat (FP), spirotetramat (STM), and fluazifop (FL).

A principle component analysis (PCA) and cluster analysis (CA) were used to visualize the dataset for the analyzed pesticides. The PCA of the measured concentrations of the 23 pesticides in citrus fruits gives nine principle compounds with eigenvalues >1. The contribution rates of PC1, PC2, PC3, PC4, PC5, PC6, PC7, PC8 and PC9 were 72.1%, 4.1%, 3.9%, 3.7%, 3.3%, 2.9%, 2.9%, 2.3% and 1.7%, respectively. The cumulative contribution of the first three principal components was 80%. The 3D scatter plot graph (Figure 2) demonstrates the segregation of certain pesticides into four different groups. The largest group included propiconazole, pyrimethanil, carbendazim, metolachlor, hexythiazox, azinphos-methyl, azoxystrobin, and prochloraz. The second group included acetamiprid, chlorpyrifos, dimethomorph, boscalid, and thiabendazole. The third group included imazalil, imidacloprid, tebufenozide, and pyrimethanil. The fourth group includes only three pesticides: picoxystrobin, etofenprox, and tebufenpyrad. PC1 showed moderate negative loading for acetamiprid and chlorpyrifos, PC2 showed strong positive loading for pyraclostrobin and imidacloprid, and PC3 showed strong positive loading for etofenprox and picoxystrobin.

**Figure 3:**
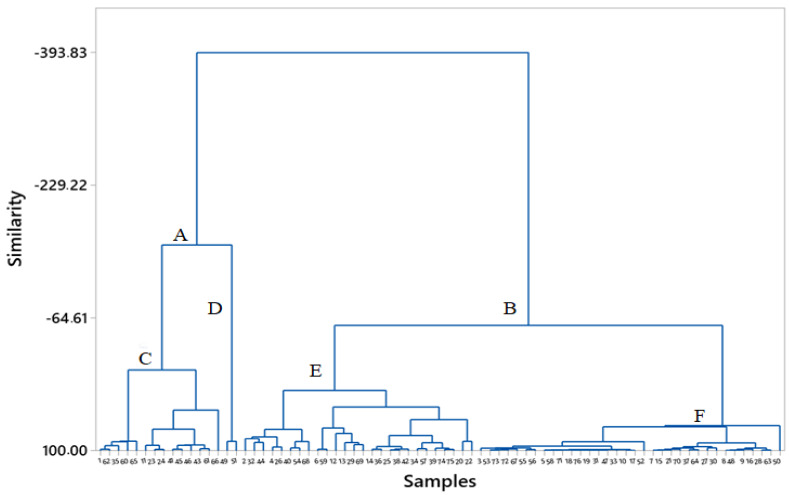
Cluster analyses of pesticide residues of citrus fruits.

The CA of the detected pesticides was performed using hierarchical cluster analysis based on the Ward linkage method and Euclidean distance matrices (Figure 3). The CA indicated two main clusters, A and B, where cluster A represents citrus fruits that contained the pesticide imazalil in higher concentrations than other samples. Samples from cluster B are characterized by either a lower concentration of the pesticide imazalil or its absence. The majority of samples from cluster A also contained other pesticides besides imazalil. Cluster A could be divided into two sub-clusters: the left (cluster C) with a higher content of imazalil (1.6 to 3.9 mg/kg) and the right (cluster D) with a lower content of imazalil (0.24 and 0.65). The residues of the pesticides thiabendazole, boscalid, and dimethomorph were also detected in the samples of cluster D. Cluster B could be divided into two sub-clusters (E and F). The left cluster E mainly comprises samples where imazalil is found in concentrations (0.030–1.6) lower than cluster A but higher than cluster F (0.010–0.46). Cluster E also forms sub-clusters: the left, in which all samples contained the pesticide thiabendazole, and the right, in which most of the samples contained pyrimethanil or azoxystrobin in addition to imazalil and other detected pesticides.

### 3.4. Risk Ranking of Pesticides in Citrus Fruits

**Figure 4:**
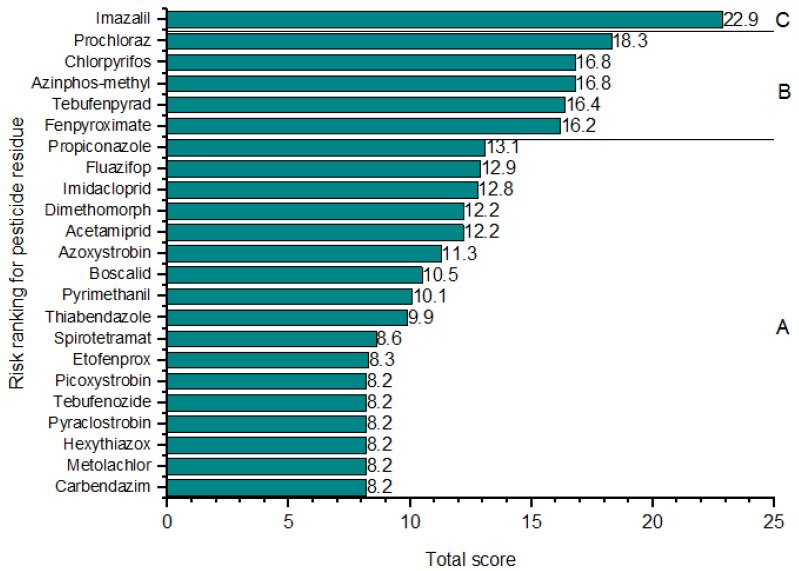
Risk ranking for the 23 detected pesticides in the citrus fruit samples. (**A**) The low-risk group, where pesticides scored below 15. (**B**) The medium-risk group, where pesticides scored from 15 to 19.9. (**C**) The high-risk group, where pesticides score at or above 20.

According to the residual risk score, the 23 detected pesticides were classified into three groups (Figure 4). Most of the detected pesticides (seventeen of 23) had residual risk scores below 15 and were considered low risk. Five pesticides (prochloraz, chlorpyrifos, azinphos-methyl, tebufenpyrad, and fenpyroximat) had risk scores from 15.0 to 19.9. They were classified as a medium-risk group, while only imazalil had a risk score at or higher than 20 and was classified into the high-risk group (residual risk score of 22.9). Imazalil was the most often detected pesticide in the analyzed fruits, with the highest detected concentrations. Based on these results, 74% of the pesticides were ranked in the low-risk group, while 22% were in the medium group.

### 3.5. Acute and Chronic Health Risk

The chronic health risk assessment (long-term intake) indicated that the risk intake values of the detected pesticides were significantly lower than the recommended ADI values. The highest mean value of chronic risk was found for imazalil (*HQ_adults_* = 0.0092 and *HQ_children_* = 0.017), which is lower than 1, indicating a low chronic risk from pesticides found in citrus fruits. The highest exposure value was also obtained for imazalil, which resulted in 0.045 for adults and 0.085 for children.

For acute health risk assessment (short-term intake), the highest exposure values were found for imazalil (*HQ_adults_* = 2.4 and *HQ_children_* = 3.8) and thiabendazole (*HQ_adults_* = 0.43 and *HQ_children_* = 1.9). The highest mean values were also obtained for these two pesticides: *HQ_adults_* = 0.19 and *HQ_children_* = 0.61 for imazalil, and *HQ_adults_* = 0.027 and *HQ_children_* = 0.10 for thiabendazole. While considering the mean exposure values for acute health risks, it can be concluded that the detected pesticides do not pose a risk in the short term. However, the exposure values above 1, obtained for several samples, suggested that exposure risks for the pesticides imazalil and thiabendazole are unacceptable in the short term, especially for children. The acute risks of imazalil for adults and thiabendazole for children were unacceptable in 3% of samples, while the acute risk of imazalil for children was unacceptable in 20% of the analyzed samples.

**Figure 5:**
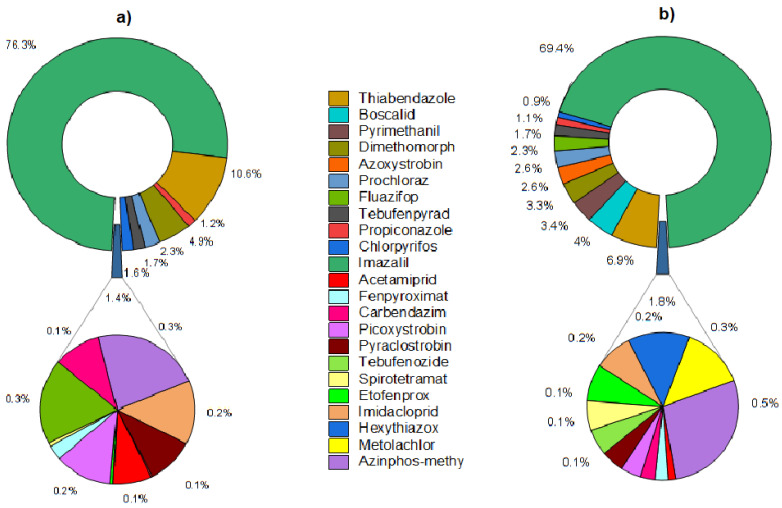
Contribution (%) of pesticide residues to *HI* for citrus fruits: (**a**) acute intake risk and (**b**) chronic intake risk for the consumer group of adults and children.

In this study, 83% of citrus fruits had multipesticide residues. Therefore, it is necessary to evaluate the cumulative risk of all pesticide residues in a single sample (Equation (11)). The contribution of individual pesticides to the total chronic/acute health risk based on the mean *HQ* values is shown in Figure 5a,b. The chronic hazard index (*HIc*) was acceptable in all samples for children and adults. The maximum value for *HIc* for adults and children are 6.3 × 10^−2^ and 7.8 × 10^−2^, respectively. Our findings are similar to studies from Poland, Brazil, and China, which also concluded that adults and children were not at risk due to long-term pesticide intake through citrus fruit consumption [10,54,55]. On the other hand, the acute hazard index (*HIa*) was unacceptable in 5.3% of the citrus fruits for adults and 26% for children. The maximum value for *HIa* for adults and children were 2.5 and 3.8, respectively.

### 3.6. Probabilistic Monte Carlo Simulation

**Figure 6:**
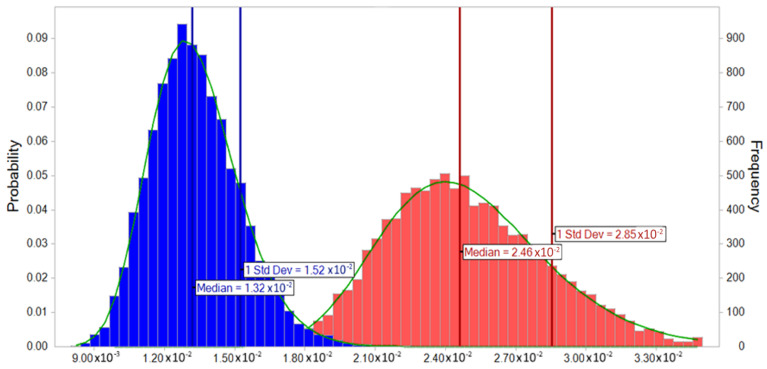
Hazard index (HI) for adults and children consumers of citrus fruit.

The Monte Carlo simulation was used for the probabilistic distribution of the cumulative chronic risk of all pesticides in citrus fruits. The probabilistic distribution of the cumulative risk for children and adults was lognormal and right-skewed (positively skewed). For the right-skewed distribution, the mean is greater than the median. Hence, it is preferable to use the median since the mean overestimates the most common values in this type of distribution. Median values for chronic *HI* in adults and children due to citrus fruit ingestion were 1.3 × 10^−2^ and 2.5 × 10^−2^ (Figure 6), respectively, which is below 1, indicating negligible long-term risk. The median value for children was higher than in adults due to the lower body weight of children. Therefore, the graph of *HI* for children has greater variation, where the standard deviation was 2.9 × 10^−2^.

**Figure 7:**
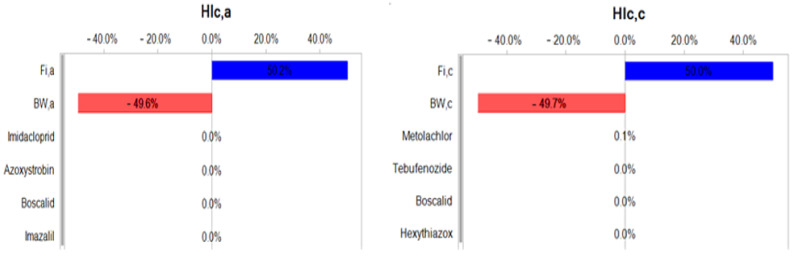
Sensitivity analysis (rank correlation) for human health risk from citrus fruit.

The sensitivity-analysis results (Figure 7) indicated that for the cumulative long-term health risk, both in adults and children, the most significantly influential factors were the ingestion rate of citrus per person (*Fi*, kg/day) and body weight (*bw*, kg). The ingestion rate positively affected the long-term risk, i.e., higher ingestion of citrus fruits contributed to higher long-term risk. On the other hand, body weight had a negative impact, where long-term risk was lower for greater body weight.

## 4. Conclusions

Twenty-three pesticides were detected in the citrus fruits purchased from Serbian markets. In all, nine pesticides were not approved by EU legislation (29% of the analyzed samples). The most detected pesticides were imazalil, azoxystrobin, dimethomorph, boscalid, pyrimethanil and thiabendazole. The highest concentrations were detected for thiabendazole and imazalil. However, the MRL values of the pesticide residues were exceeded for imazalil, propiconazole, azinphos-methyl, dimethomorph, prochloraz, chlorpyrifos, picoxystrobin, and fluazifop. Although most of the samples had multiple residues, and 28% contained pesticides at or above maximum residue levels (MRLs), the risk ranking method indicated that 74% of the detected pesticides posed a low risk to human health. Furthermore, the health risk assessment revealed negligible chronic health risks, while acute risks were unacceptable in several samples due to the presence of imazalil and thiabendazole. The findings of this study support the need for pesticide residue monitoring in citrus fruits and health risk assessments to determine whether and to what extent they affect human health.

## Figures and Tables

**Table 1:** Correlation matrix for pesticide residues in citrus fruits. Imazalil (IMA), thiabendazole (TBZ), propiconazole (PRP), pyrimethanil (PYM), carbendazim (CRB), metolachlor (MTLC), hexythiazox (HXTZ), azinphos-methy (AZM), azoxystrobin (AZX), boscalid (BOS), imidacloprid (IMD), dimethomorph (DMM), prochloraz (PRO), tebufenpyrad (TEBU), pyraclostrobin (PYR), tebufenozide (TBF), acetamiprid (ACP), chlorpyrifos (CPF), etofenprox (EFP), picoxystrobin (PC), fenpyroximat (FP), spirotetramat (STM), and fluazifop (FL).

	IMA	TBZ	PRP	PYM	CRB	MTLC	HXTZ	AZM	AZX	BOS	IMD	DMM	PRO	TEBU	PYR	TBF	ACP	CPF	EFP	PC	FP	STM
TBZ	−0.035																					
PRP	−0.051	−0.052																				
PYM	−0.069	−0.005	0.048																			
CRB	0.136	−0.026	−0.027	−0.062																		
MTLC	−0.048	−0.041	−0.028	−0.064	−0.026																	
HXTZ	−0.085	−0.049	−0.028	−0.018	−0.026	−0.027																
AZM	−0.043	−0.049	−0.028	−0.064	−0.026	−0.027	−0.027															
AZX	0.073	−0.068	−0.004	−0.085	−0.041	−0.042	−0.042	0.319														
BOS	0.251	0.081	−0.059	−0.004	−0.054	−0.056	−0.026	−0.056	0.014													
IMD	0.179	0.018	−0.043	−0.001	−0.040	−0.041	−0.041	−0.041	−0.034	−0.055												
DMM	−0.003	0.529	−0.092	−0.045	−0.086	−0.089	−0.088	−0.089	−0.079	0.269	−0.083											
PRO	0.094	−0.057	−0.032	−0.074	−0.030	−0.031	−0.031	−0.031	0.001	−0.053	−0.048	0.170										
TEBU	−0.116	0.082	−0.025	−0.058	−0.024	−0.024	−0.024	−0.024	−0.038	0.033	0.302	0.061	−0.028									
PYR	0.286	−0.003	−0.025	−0.057	−0.023	−0.024	−0.024	−0.024	−0.013	−0.023	0.743	−0.033	−0.028	0.269								
TBF	0.167	−0.049	−0.028	0.086	−0.026	−0.027	−0.026	−0.027	−0.027	−0.056	0.367	−0.088	−0.031	−0.024	0.541							
ACP	−0.008	0.079	−0.020	0.157	−0.018	−0.019	−0.019	−0.019	−0.030	0.490	−0.029	0.312	−0.022	−0.017	−0.017	−0.019						
CPF	−0.050	0.047	−0.028	0.107	−0.026	−0.026	−0.026	−0.026	−0.008	0.388	−0.041	0.226	−0.030	−0.024	−0.023	−0.026	0.830					
EFP	−0.090	−0.035	−0.020	−0.045	−0.018	−0.019	−0.019	−0.019	−0.030	−0.040	−0.029	−0.062	−0.022	0.331	−0.017	−0.019	−0.013	−0.019				
PC	−0.090	−0.035	−0.020	−0.045	−0.018	−0.019	−0.019	−0.019	−0.030	−0.040	−0.029	−0.062	−0.022	0.331	−0.017	−0.019	−0.013	−0.019	0.999			
FP	0.013	−0.035	−0.020	−0.036	−0.018	−0.019	−0.019	−0.019	−0.012	−0.009	−0.029	0.035	−0.022	−0.017	−0.017	−0.019	−0.013	−0.019	−0.013	−0.013		
STM	0.138	−0.049	−0.028	−0.063	−0.026	−0.027	−0.026	−0.027	0.027	−0.001	0.000	−0.021	0.091	−0.024	−0.024	−0.026	−0.019	−0.026	−0.019	−0.019	−0.019	
FL	−0.087	0.039	−0.028	0.279	−0.026	−0.027	−0.027	−0.027	−0.032	−0.006	−0.041	0.100	−0.031	−0.024	−0.024	−0.027	−0.019	−0.026	−0.019	−0.019	−0.019	−0.027

## Data Availability

The data that support the findings of this study are available from the corresponding author upon reasonable request.

## Supplementary Materials

The following supporting information can be downloaded at: https://www.mdpi.com/article/10.3390/foods12132454/s1, Table S1. Definition and individual scores of indices for the pesticide residual risk ranking; Table S2. Summary table for LD_50_, ADI, MRL, ARfD values and assigned scores for indices A and B. The LD_50_ values are adopted from the WHO database [41]. Most of the ADI values are sourced from EU Pesticide database [42], and those values that did not exist in this database were taken from JMPR database [43] or from Pesticide Properties DataBase (PPDB) [44]. The MRLs values were sourced from EU pesticide database [20]. The ARfD values were taken from EU pesticide database [20] or PPDB [44]; Table S3. Assigned scores for indices A-F; Table S4. Descriptive statistics for detected pesticides in citrus fruits.
