# Supplementary material for: Multivariate Assessment and Risk Ranking of Pesticide Residues in Citrus Fruits
Source: Foods. 2023 Jun 22;12(13):2454. doi: 10.3390/foods12132454 (PMC10340182; doi:10.3390/foods12132454)
Supplement: Supplementary file 1 [file foods-12-02454-s001.zip › foods-2437908-supplementary.pdf]

Supplementary Materials

Multivariate Assessment and Risk Ranking of Pesticide Residues in Citrus Fruits

Jelena Radulović [1], Milica Lučić [2], Aleksandra Nešić [3] and Antonije Onjia [4,*]

[1] Anahem Laboratory, Mocartova 10, 11160 Belgrade, Serbia
[2] Innovation Center of the Faculty of Technology and Metallurgy, Karnegijeva 4, 11120 Belgrade, Serbia
[3] Vinča Institute of Nuclear Sciences, University of Belgrade, Mike Alasa 12-14, Belgrade, Serbia
[4] Faculty of Technology and Metallurgy, University of Belgrade, Karnegijeva 4, 11120 Belgrade, Serbia
* Correspondence: onjia@tmf.bg.ac.rs

Table S1. Definition and individual scores of indices for the pesticide residual risk ranking.

Index	Item	Definition	Score	Definition	Score	Definition	Score	Definition	Score
A	Toxicity (mg/kg)	Low	2	Moderate	3	High	4	Hypertoxic	5
B	Potency (mg/kg)	$>10^{-2}$	0	$>10^{-4}$-10^{-2}	1	$>10^{-6}$-10^{-4}	2	$<10^{-6}$	3
C	Proportion of diet (%)	<2.5	0	2.5-20	1	20-50	2	50-100	3
D	Frequency of dosing (%)	<2.5	0	2.5-20	1	20-50	2	50-100	3
E	Evidence for high exposure groups	No	0	Unlikely	1	Likely	2	Existing	3
F	Residue level (mg/kg)	ND	1	<MRL	2	≥1-10 MRL	3	≥10 MRL	4

Table S2. Summary table for LD_{50}, ADI, MRL, ARfD values and assigned scores for indices A and B. The LD_{50} values are adopted from the WHO database [1]. Most of the ADI values are sourced from EU Pesticide database [2], and those values that did not exist in this database were taken from JMPR database [3] or from Pesticide Properties DataBase (PPDB) [4]. The MRLs values were sourced from EU pesticide database [5]. The ARfD values were taken from EU pesticide database [5] or PPDB [4].

No	Pesticide	LD_{50} - JMPR WHO database [1]	A	ADI - EU Pesticide database [2]	B	MRL - EU Pesticide database [5]	ARfD - EU Pesticide database [2]
		(mg/kg)		(mg/kg/day)		(mg/kg),	mg/kg bw
1	Imazalil	227	3	0.025	0	4 Grapefruits and oranges; 5 lemons, limes, and mandarins; 0.01 Others	0.05
2	Thiabendazole	3330	2	0.1	0	7	0.1
3	Propiconazole	1520	3	0.04<br>0.07 [3]	0	0,01	0.1
4	Pyrimethanil	4150	2	0.17	0	8	Not Applicable
5	Carbendazim	>10000	2	0.02<br>0.03 [3]	0	0.2 Grapefruit and Oranges; 0,7 lemons, limes, and mandarins; 0.1 Others	0.02
6	Metolachlor	2780	2	0.1 PPDB [4]	0	0.05	
7	Hexythiazox	>5000	2	0.03	0	0.5	Not Applicable
8	Azinphos-methy	16	4	0.03 [3]	0	0.01	0.1[3]
9	Azoxystrobin	>5000	2	0.2	0	15	Not Applicable
10	Boscalid	>5000	2	0.04	0	2	Not Applicable
11	Imidacloprid	450	3	0.06	0	0.9	0.08
12	Dimethomorph	3500	2	0.05	0	0.01 All. 0.8 Oranges	0.6
13	Prochloraz	1600	3	0.01	1	0.03	0.025
14	Tebufenpyrad	595	3	0.01	1	0.6	0.02
15	Pyraclostrobin	>2000	2	0.03	0	2	0.03
16	Tebufenozide	>5000	2	0.02	0	2	0.9[3]
17	Acetamiprid	c140	3	0.025	0	0.9	0.025
18	Chlorpyrifos	135	3	0.01 [3]	1	0.01	0.005 [4]
19	Etofenprox	>10000	2	0.03	0	1.5	1
20	Picoxystrobin	D>2000	2	0.09 [3]	0	0.01	0.09 [3]
21	Fenpyroximat	245	3	0.01	1	0.5	0.02
22	Spirotetramat	>2000	2	0.05	0	0.5	1
23	Fluazifop	2451	2	0.004 [3]	1	0.01	0.4 [3]

1. *The WHO Recommended Classification of Pesticides by Hazard and Guidelines to Classification*; 2019 edition.; Geneva, 2020; ISBN 978-92-4-000566-2.
2. European Commission EU Pesticides Database - Active Substances Available online: https://ec.europa.eu/food/plant/pesticides/eu-pesticides-database/start/screen/active-substances (accessed on 10 April 2023).
3. WHO Inventory of Evaluations Performed by the Joint Meeting on Pesticide Residues (JMPR). Available online: https://apps.who.int/pesticide-residues-jmpr-database/Home/Range/All (accessed on 8 December 2022).
4. Agriculture & Environment Research Unit (AERU) at the University of Hertfordshire PPDB - Pesticide Properties Database Available online: http://sitem.herts.ac.uk/aeru/ppdb/en/index.htm (accessed on 12 April 2023).
5. European Commission EU Pesticides Database - MRLs Available online: https://ec.europa.eu/food/plant/pesticides/eu-pesticides-database/start/screen/mrls (accessed on 12 April 2023).

Table S3. Assigned scores for indices A-F.

Pesticide	Toxicity score LD50 A	Potency score ADI B	Proportion of diet (%) score C	Frequency of dosing (%) score D	Evidence for high exposure groups score E	Residue level score F
Imazalil	3	0	0	1	3	1,908
Thiabendazole	2	0	0	1	3	1,237
Propiconazole	3	0	0	1	3	1,092
Pyrimethanil	2	0	0	1	3	1,263
Carbendazim	2	0	0	1	3	1,026
Metolachlor	2	0	0	1	3	1,026
Hexythiazox	2	0	0	1	3	1,026
Azinphos-methyl	4	0	0	1	3	1,053
Azoxystrobin	2	0	0	1	3	1,408
Boscalid	2	0	0	1	3	1,316
Imidacloprid	3	0	0	1	3	1,066
Dimethomorph	2	0	0	1	3	1,526
Prochloraz	3	1	0	1	3	1,145
Tebufenpyrad	3	1	0	1	3	1,026
Pyraclostrobin	2	0	0	1	3	1,026
Tebufenozide	2	0	0	1	3	1,026
Acetamiprid	3	0	0	1	3	1,013
Chlorpyrifos	3	1	0	1	3	1,053
Etofenprox	2	0	0	1	3	1,039
Picoxystrobin	2	0	0	1	3	1,026
Fenpyroximat	3	1	0	1	3	1,013
Spirotetramat	2	0	0	1	3	1,079
Fluazifop	2	1	0	1	3	1,079

Table S4. Descriptive statistics for detected pesticides in citrus fruits.

Pesticide	No. of samples with pesticide residue	Max (mg/kg)	Min (mg/kg)	Mean (mg/kg)	Median (mg/kg)	MRL (mg/kg)	Detection (%)
Imazalil	67	3.9	0.01	0.9	0.52	4.0 Grapefruits and oranges; 5.0 lemon, limes and mandarinas; 0.01 Others	88
Thiabendazole	18	5.9	0.09	1.2	0.85	7	24
Propiconazole	3	0.21	0.04	0.11	0.09	0,01	4.0
Pyrimethanil	20	1.3	0.02	0.41	0.32	8	26
Carbendazim	2	0.05	0.03	0.04	0.04	0.2 Grapefruit and Oranges; 0.7 lemons, limes and mandarinas; 0.1 Others	2.6
Metolachlor	2	0.02	0.02	0.02	0.02	0.05	2.6
Hexythiazox	2	0.03	0.02	0.03	0.03	0.5	2.6
Azinphos-methyl	2	0.02	0.02	0.02	0.02	0.01	2.6
Azoxystrobin	31	1.5	0.02	0.12	0.04	15	41
Boscalid	25	0.61	0.02	0.1	0.04	2.0	33
Imidacloprid	5	0.04	0.02	0.02	0.02	0.9	6.6
Dimethomorph	28	0.48	0.02	0.16	0.13	0.01 All. 0.8 Oranges	37
Prochloraz	4	1.2	0.15	0.55	0.42	0.03	5.3
Tebufenpyrad	2	0.14	0.05	0.1	0.1	0.6	2.6
Pyraclostrobin	2	0.11	0.04	0.07	0.07	2.0	2.6
Tebufenozide	2	0.03	0.02	0.03	0.03	2.0	2.6
Acetamiprid	1	0.09	0.09	0.09	0.09	0.9	1.3
Chlorpyrifos	2	0.08	0.05	0.07	0.07	0.01	2.6
Etofenprox	1	0.02	0.02	0.02	0.02	1.5	1.3
Picoxystrobin	1	0.03	0.03	0.03	0.03	0.01	1.3
Fenpyroximat	1	0.01	0.01	0.01	0.01	0.5	1.3
Spirotetramat	3	0.08	0.01	0.04	0.03	0.5	4.0
Fluazifop	2	0.48	0.43	0.46	0.46	0.01	2.6
